# Oxa-noribogaine reduces alcohol drinking through aversion learning and by altering glutamatergic activity in the mPFC

**DOI:** 10.21203/rs.3.rs-9103509/v1

**Published:** 2026-03-31

**Authors:** Marcus Meinhardt, Ivan Skorodumov, Florian Walter, Merve Akan, Tobias Buchborn, Yéléna LE PRIEULT, Marvin Urban, Rainer Spanagel, Livia von Ammon, Carsten Hopf, Liubov Kalinichenko, Christian Mueller, Christine Winter, Ravit Hadar, Asude Zülal Gül, Ben Massuda, Maj Hildebrandt, Esi Domi, Adana Keshishian, Roberto Ciccocioppo, Dalibor Sames, Vaclav Havel, Leah Woods, Angela Beeson

**Affiliations:** Central Institute for Mental Health; Central Institute for Mental Health; Central Institute for Mental Health; Central Institute for Mental Health; Central Institute for Mental Health; Central Institute for Mental Health; Central Institute for Mental Health; Institute of Psychopharmacology, Central Institute of Mental Health, Mannheim, Germany; HS Mannheim; Technische Hochschule Mannheim; Friedrich-Alexander-University of Erlangen-Nürnberg; University of Erlangen-Nuremberg; Freie Universität Berlin; Charite; Charite; Charite; University of Camerino; University of Camerino; University of Camerino; Columbia University; Columbia University; 8Wake Forest University School of Medicine; 8Wake Forest University School of Medicine

**Keywords:** Alcohol use disorder, Psychedelic-inspired therapeutics, Oxa-noribogaine, Context-dependent learning, Cortical plasticity, Glutamatergic signaling, Neurotrophic regulation

## Abstract

Alcohol use disorder is a major global health problem, and current treatments often fail to produce lasting reductions in harmful drinking1,2. Psychedelic-assisted therapies may promote durable behavioural change by enhancing brain plasticity during emotionally meaningful experiences, but progress has been limited by a lack of experimental models that capture these context-dependent effects3,4. Here we show that the ibogaine-derived compound oxa-noribogaine reduces alcohol consumption by strengthening learning from negative drinking outcomes in translational rat models of alcohol dependence. The compound produces sustained decreases in alcohol intake and relapse-like drinking, matches or exceeds the efficacy of its parent compound ibogaine, and does so without detectable motor or cardiac liabilities. These behavioural effects are associated with transient changes in prefrontal brain activity, lasting alterations in glutamatergic signalling after aversion-related learning, and normalization of neurotrophic signalling in cortico-striatal circuits. The therapeutic effects generalize across several translational models, genetically diverse animals and independent study sites. Together, these findings identify oxa-noribogaine as a promising and potentially safer treatment candidate for alcohol use disorder. More broadly, the results establish a preclinical framework for studying psychedelic-inspired therapies that harness context-dependent neuroplasticity to reduce compulsive substance use and support adaptive behavioural change.

Alcohol use disorder (AUD) remains a major global health burden, affecting more than 400 million people worldwide and accounting for approximately 2.6 million deaths annually^2^. Despite decades of research, pharmacological treatment options remain limited with modest efficacy and high non-response rates^5^. The slow pace of therapeutic innovation underscores the urgent need for mechanistically novel approaches.

Psychedelic compounds have reemerged as promising treatments for several psychiatric disorders, including addiction^6,7^. Among these, ibogaine, a naturally occurring indole alkaloid, has shown anti-addictive effects across multiple substances^8^. However, its clinical use is restricted by cardiotoxicity due to human ether-à-go-go-related (hERG) potassium channel inhibition^9,10^. Oxa-noribogaine, a recently developed analogue, retains ibogaine’s pharmacological profile while lacking cardiotoxic effects and reduces opioid self-administration in preclinical models, positioning it as a promising candidate for AUD treatment^11^. A central translational challenge in psychedelic research lies in modelling the therapeutic context, particularly in addiction models^12–15^. In humans, psychedelic administration occurs ideally within structured psychotherapeutic frameworks, where subjective “breakthrough” experiences and confrontation with maladaptive behaviours are considered critical for clinical efficacy^3,16^. Such context-dependent and introspective components – often described as “set and setting” – are inherently difficult to reproduce in animal models, potentially limiting translational validity^4^.

To address this gap, we developed a novel aversive alcohol-paired learning paradigm in rodents designed to model the confrontation with negative consequences that characterizes psychedelic-assisted therapy. This paradigm pairs alcohol self-administration with aversive experiential learning, thereby introducing a psychologically meaningful context into preclinical addiction research. We hypothesized that combining this aversion-based framework with oxa-noribogaine treatment would produce sustained reductions in excessive alcohol consumption.

At the circuit level, chronic alcohol use is associated with impaired medial prefrontal cortex (mPFC) control over mesolimbic reward pathways, including glutamatergic projections to the nucleus accumbens and amygdala^17,18^. Alcohol-induced dysregulation of glutamatergic signalling and neurotrophic pathways, including brain-derived neurotrophic factor (BDNF) and glial cell line-derived neurotrophic factor (GDNF), contributes to compulsive intake^19,20^. Notably, ibogaine and oxa-noribogaine modulate GDNF expression in the ventral tegmental area (VTA), a mechanism implicated in reduced drug consumption^21,11^. Building on these observations, we proposed that oxa-noribogaine would reduce alcohol intake by restoring prefrontal control and restoring neurotrophic signalling within addiction-relevant circuits. We applied a multimodal approach (Extended Data Fig. 1) that integrated medicinal chemistry, receptor pharmacology, behavioural modelling using our aversive alcohol-paired paradigm, and systems-level neurobiological analyses to define its mechanism of action and therapeutic potential in AUD.

## Oxa-noribogaine enhances aversion-paired alcohol exposure and reduces subsequent alcohol self-administration

**Figure 1:**
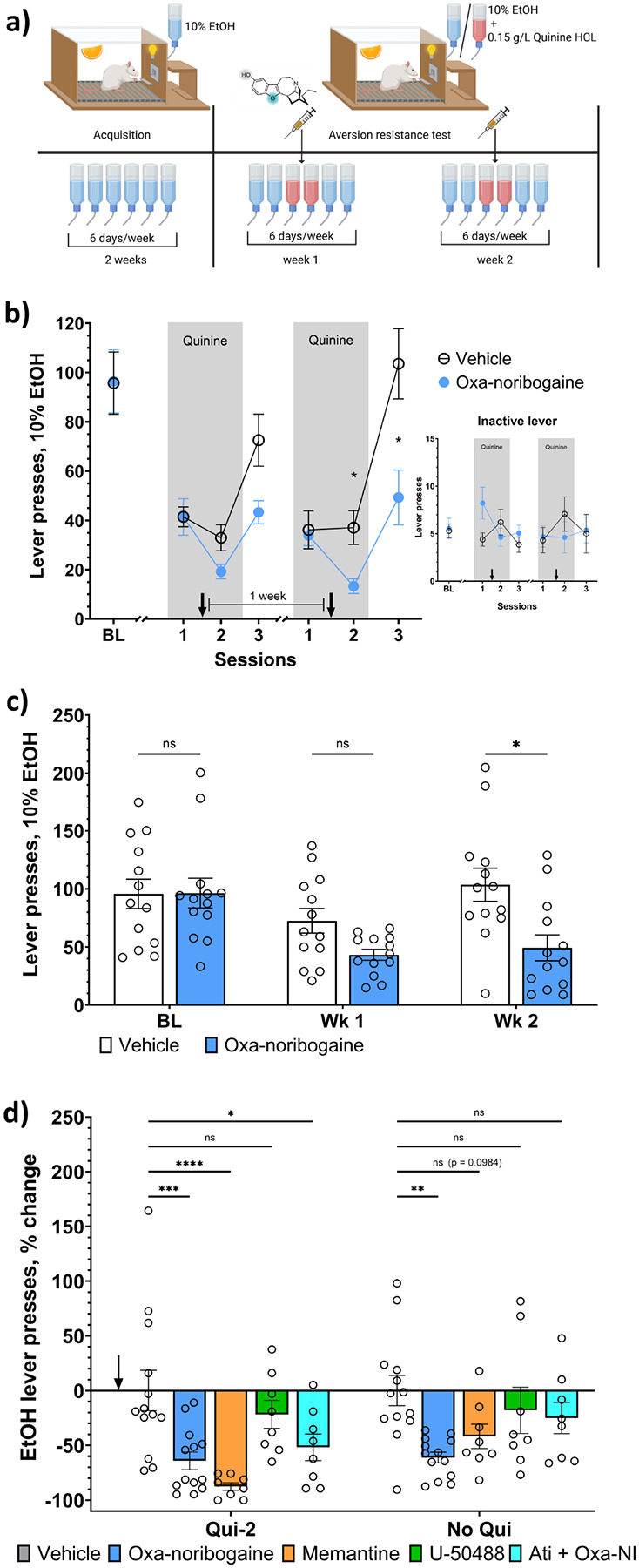
Efficacy of oxa-noribogaine on alcohol self-administration following aversion-paired alcohol exposure. a) Schematic representation of experiment design. Orange slice = presence of smell cue (orange oil) signalling reward availability, electric bulb = blinking light (3 sec) during reward delivery. Blue bottle = one 30-min operant session with 10% EtOH, red bottle = 0.15 g/L quinine added to 10% EtOH. Syringe = application of vehicle / oxa-noribogaine. b) Oxa-noribogaine augments aversion learning by quinine-paired alcohol exposure and reduces subsequent quinine-free alcohol self-administration without affecting motor activity. Presses on the inactive lever (inset) were recorded but no reward was delivered. BL = baseline (average reward lever presses of the last 3 days of acquisition phase), 1–2 = quinine-adulterated sessions, 3 = first quinine-free session. Arrows indicate when vehicle (n = 13) or oxa-noribogaine (n = 13) was administered (i.p.). c) Self-administration of non-adulterated alcohol is suppressed by oxa-noribogaine in the first (WK1) as well as second week (WK2) after the aversion (quinine)-paired alcohol exposure. d) NMDA receptor antagonism and KOR agonism both contribute to the effect of oxa-noribogaine. Change in reward lever presses expressed as % of vehicle group average on a given day. Qui-2 = second quinine-adulterated session, No Qui = first quinine-free session after the second quinine session. Arrow indicates when vehicle (n = 13), oxa-noribogaine (n = 13), memantine (n = 8), U-50488 (n = 8) or aticaprant + oxa-noribogaine (n = 8) was administered (i.p.). NMDAR antagonist memantine (25 mg/kg) suppresses quinine-adulterated alcohol intake, but the effect is not carried over into the next day session with quinine-free alcohol. Pretreatment with KOR antagonist Aticaprant (1 mg/kg) produces a smaller effect compared to oxa-noribogaine alone that also does not last into the quinine-free session. KOR agonist U-50488 (10 mg/kg) fails to reduce quinine-adulterated and quinine-free alcohol self-administration. Data are presented as mean ± SEM. Statistical tests used: Two-way repeated measures ANOVA, post-hoc Sidak’s (b-c) or Dunnett’s (d) multiple comparisons test, *P<0.05, **P<0.01, ***P<0.001, ****P<0.0001.

Rats were trained to self-administer 10% ethanol in daily 30-min operant sessions for 12 days using odor- and light-cued reinforcement (Fig. 1a, extended data Fig. 2b). To model aversion-paired alcohol exposure, ethanol was adulterated with quinine. A pilot dose–response analysis identified 0.15 g/L quinine hydrochloride as the lowest concentration reliably producing strong aversion, and this concentration was used for all subsequent experiments (Extended Data Fig. 2a). Following stable self-administration, rats underwent two aversion cycles separated by renewed alcohol access (Fig. 1a). Baseline responding was defined as the mean of the three sessions preceding aversion and did not differ between groups prior to treatment (vehicle: mean 95.7 ± 45.5, oxa-noribogaine: mean 96.4 ± 46.1). Oxa-noribogaine (30 mg/kg, i.p.^11^, followed by 20 mg/kg 3 h later) or vehicle (aqua, i.p.) was administered after the first quinine session. This injection schedule was designed to compensate for the much faster metabolic clearance of oxa-noribogaine in rats compared to humans. Through this repeated-dosing approach, the overall exposure to the compound is increased to more closely approximate the human condition, modelled on ibogaine, where the substantially slower metabolism allows ibogaine and its active metabolites to remain in the system for extended periods – often sustaining the ibogaine experience for up to 72 hours^22–25^.

Quinine adulteration robustly suppressed alcohol responding in both groups during aversion sessions (Fig. 1b). After the first aversion cycle, oxa-noribogaine-treated rats showed a trend toward reduced alcohol intake relative to controls, although this did not reach statistical significance (p = 0.0645, Fig. 1b,c). Upon re-exposure to unadulterated ethanol, control animals rebounded toward baseline intake (78%), whereas oxa-noribogaine-treated rats exhibited a blunted recovery (44.9%). Following the second aversion cycle, oxa-noribogaine produced a significant and sustained reduction in alcohol self-administration (F(1,24) = 5.25, p = 0.031; Hedges’ g = 1.14, 95% CI: 0.31–1.97), whereas control animals returned to baseline drinking levels (Fig. 1b,c). In contrast, oxa-noribogaine-treated rats maintained significantly lower responding during subsequent quinine-free sessions (p = 0.0448, post-hoc Sidak’s test). Inactive lever presses (Fig. 1b) were unaffected, indicating preserved motor function and behavioural specificity. Notably, the divergence between groups increased from the first to the second aversion cycle, suggesting that oxa-noribogaine enhances aversion-based learning rather than acutely suppressing alcohol consumption. The persistence of reduced intake for at least 48 h after drug administration – when alcohol no longer contained quinine – supports the interpretation that oxa-noribogaine strengthens the encoding or consolidation of aversive alcohol-associated memories (Fig. 1c).

## NMDA receptor inhibition and KOR activation partially contribute to the effect of oxa-noribogaine on aversion learning and subsequent alcohol self-administration

Iboga alkaloids exhibit a complex “matrix pharmacology”^26^, engaging multiple central targets including for example κ-opioid receptors (KOR), monoamine transporters, and N-methyl-D-aspartate receptors (NMDARs, Extended Fig. 1). Two of these systems are particularly relevant to alcohol use disorder. Ethanol itself inhibits NMDARs^27^, and NMDAR antagonists such as memantine reduce ethanol self-administration in rodents, potentially via partial substitution for alcohol’s interoceptive effects^28^. In parallel, the dynorphin/KOR system regulates aversive-affective processing, and oxa-noribogaine displays high agonist potency at KOR (EC50 = 43 nM, G protein activation) alongside enhanced NMDAR inhibition relative to noribogaine (IC50 = 24 μM vs. 42 μM, radioligand displacement assay)^11^.

To assess the contribution of these targets to the behavioural effects observed in our aversion-paired paradigm, we first mapped oxa-noribogaine brain distribution using spatial MALDI mass spectrometry imaging (MALDI-MSI). The compound accumulated predominantly in prelimbic and infralimbic medial prefrontal cortex and hippocampus, with lower levels in ventral tegmental area and brainstem nuclei. This regional profile closely overlapped with known NMDAR abundance (Extended Data Fig. 3a-d, Supplementary Table 1, MALDI-MSI validation in supplementary Information). Time course quantification of relative ion intensities of oxa-noribogaine revealed robust brain exposure at 30 minutes post-injection and a reduced yet still significant presence at 4 hours, whereas no detectable signal remained at 24 hours (Extended Data Fig. 3e-f).

We then performed comparative pharmacology experiments using the selective NMDAR antagonist memantine (25 mg/kg)^29^, the selective KOR agonist U-50488 (10 mg/kg)^30^, and oxa-noribogaine with or without the KOR antagonist aticaprant (1 mg/kg)^11^. During the second quinine session, both oxa-noribogaine (F (4, 45) = 7.48, p = 0.0001; second quinine session p = 0.0007, Dunnett’s post-hoc test; Hedges’ g = 1.21, 95% CI: 0.38–2.05) and memantine (p = 0.0001, Hedges’ g = 1.58, 95% CI: 0.58–2.59) significantly reduced alcohol responding relative to controls, whereas U-50488 alone (p = 0.6262, Hedges’ g = 0.37, 95% CI: −0.52–1.25) had no effect (Fig. 1d). Pretreatment with aticaprant attenuated the behavioural effect of oxa-noribogaine, suggesting KOR involvement (p = 0.0262, Hedges’ g = 0.88, 95% CI: −0.04–1.8). Critically, 48 h later – after quinine removal – only oxa-noribogaine-treated animals maintained significantly reduced alcohol self-administration (p = 0.0014, Hedges’ g = 1.14, 95% CI: 0.31–1.97). Neither memantine (p = 0.0984, Hedges’ g = 0.92, 95% CI: −0.01–1.84), U-50488 (p = 0.7634, Hedges’ g = 0.32, −0.56–1.21), nor the aticaprant/oxa-noribogaine combination (p = 0.5054, Hedges’ g = 0.52, 96% CI: −0.38–1.41) produced sustained suppression.

Together, these findings indicate that NMDAR antagonism can acutely enhance aversion-related suppression of alcohol intake, while KOR signalling contributes to oxa-noribogaine’s behavioural effects. However, neither mechanism alone was sufficient to reproduce the persistent reduction in alcohol seeking. The durable impact of oxa-noribogaine therefore appears to arise from its combined, multi-receptor “matrix pharmacology” rather than from isolated target engagement.

## Effects of oxa-noribogaine on glutamatergic activity in the mPFC

**Figure 2:**
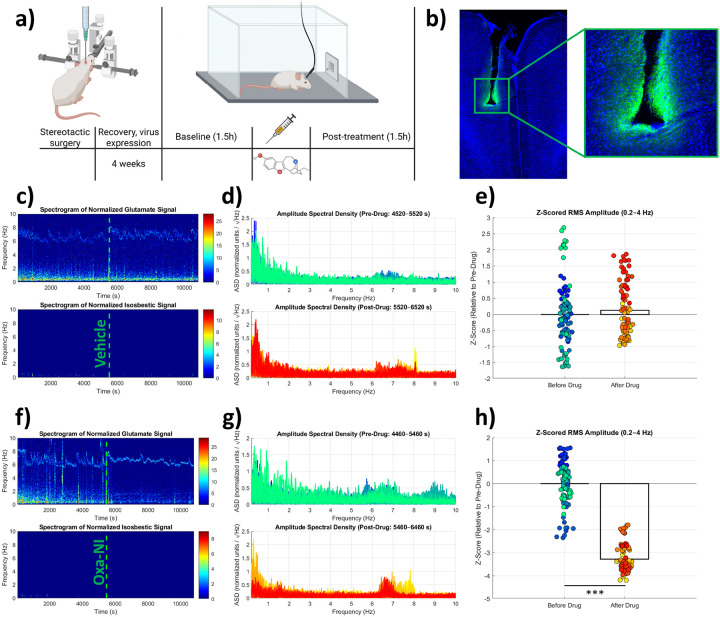
Acute effects of oxa-noribogaine on glutamatergic activity in the mPFC. a) Schematic representation of the experiment design. b) anti-GFP (green) and DAPI (blue) immunostaining of mPFC, confirming the virus injection- and fiber placement site. Scale: 10x. Spectrogram of normalized glutamate (top) or isosbestic (bottom) signal from the representative animal after vehicle c) or oxa-noribogaine f) treatment. The vertical dashed line indicates the time of injection. Coloured traces represent 100-second sliding windows (advanced every 10 s); this colour scheme is used consistently across graphs (c-h). Amplitude Spectral Densities (ASD) of the glutamate signal across sliding windows from c) and f) after vehicle d) or oxa-noribogaine g) treatment. RMS amplitude (root mean square) of the time-domain glutamate signal (0.2–4 Hz bandpass filtered; z-scored to baseline), computed for each sliding window shown in c)/f) after vehicle e) or oxa-noribogaine h) treatment. Unpaired Mann-Whitney (e, h) test. ***P<0.001.

Glutamatergic signalling in the mPFC is critically involved in chronic alcohol consumption^17,18^. To determine whether oxa-noribogaine modulates mPFC glutamate dynamics *in vivo*, we performed fiber photometry using viral expression of the circularly permuted green fluorescence protein (cpGFP)-based fluorescent sensor iGluSnFR, sampling at 20 Hz in awake, freely moving rats (Fig. 2a,b). Following a 90-min baseline recording, animals received vehicle or oxa-noribogaine (30 mg/kg, i.p.), and glutamate signals were monitored for additional 90 min.

Oxa-noribogaine induced a rapid and pronounced desynchronization of mPFC glutamatergic activity. Relative to vehicle, treated animals exhibited reduced amplitude spectral density (ASD) (t = 8.607, df = 180, p < 0.0001, Hedges’ g = 1.27, 95% CI: 0.95–1.59) and root mean square (RMS) signal amplitude (t = 8.941, df = 180, p < 0.0001, Hedges’ g = 1.32, 95% CI: 1.00–1.64), accompanied by increased Shannon entropy (t = 5.764, df = 180, p < 0.0001, Hedges’ g = −0.85, 95% CI: −1.15–(−0.55)) and a shift toward higher peak frequencies (t = 8.667, df = 180, p < 0.0001, Hedges’ g = −1.28, 95% CI: −1.6–(−0.96)) (Fig. 2c–h, Extended Data Fig.5 a-d). Comparing baseline and post-injection periods in the same animals revealed significant oxa-noribogaine–induced reductions in normalized area under the curve (AUC) of ASD (paired t-test: t = 4.184, df = 4, p = 0.0139) and RMS amplitude (t = 3.633, df = 4, p = 0.0221), as well as significant increases in Shannon entropy (t = 3.089, df = 4, p = 0.0366) and normalized peak frequency (t = 3.259, df = 4, p = 0.0311) (Extended Data Fig. 5e-h). Importantly, mass spectrometry measurements revealed no changes in total glutamate or glutamine levels in the mPFC, indicating that the observed effects reflect altered temporal organisation rather than altered neurotransmitter abundance (Extended data Fig. 4a-h). Together, these results demonstrate that oxa-noribogaine acutely disrupts the temporal organisation and spectral structure of glutamatergic signalling in the mPFC, consistent with a rapid desynchronization of cortical glutamate dynamics.

**Figure 3:**
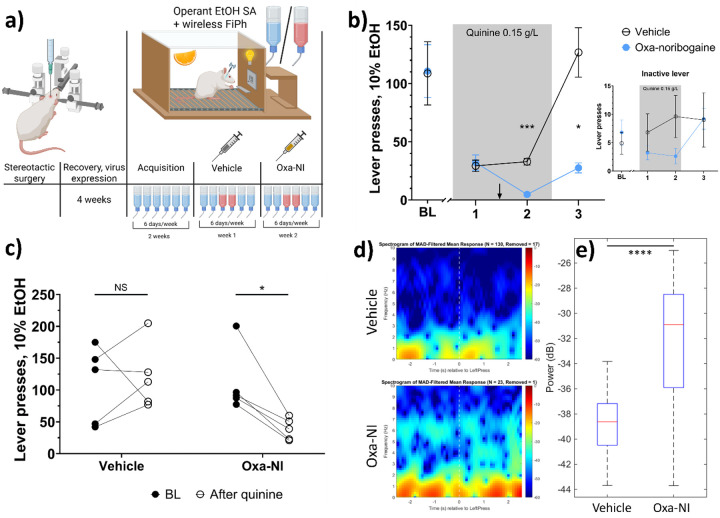
Glutamatergic activity is enhanced in mPFC the next day after the injection. a) Adapted experiment design for wireless fiber photometry; icons are the same as on fig.1.; n = 5. b) Oxa-noribogaine reduces quinine-free alcohol self-administration also in crossover-design experiment. c) Within-subject effects of vehicle or oxa-noribogaine on quinine-free alcohol self-administration (day 3). d) Spectrogram of mean absolute deviation (MAD) filtered glutamate signal power surrounding the event of reward lever press during quinine-free session; top – after vehicle (n = 130 lever presses), bottom – after oxa-noribogaine (n = 21 lever presses. e) Enhanced power of glutamate oscillations 48h after administration of oxa-noribogaine. Statistical tests used: repeated-measures two-way ANOVA, post-hoc Sidak’s multiple comparisons test (b); paired Student’s t-test (c); paired Wilcoxon test (e). Abbreviations: NS = not significant, *P<0.05, **P<0.01, ***P<0.001, ***P<0.0001.

We next asked how these dynamics evolve during aversion-paired alcohol self-administration. Using wireless fiber photometry, we recorded mPFC glutamate activity in rats undergoing the quinine-based aversion paradigm. To enable within-subject comparison, animals received vehicle during the first aversion cycle and oxa-noribogaine during the second (Fig. 3a, cross-over design). Consistent with earlier behavioural results, oxa-noribogaine enhanced aversion learning and reduced alcohol self-administration after quinine removal (interaction: F(1, 8) = 6.089, p = 0.0389; treatment effect: t = 2.984, df = 8, p = 0.0347; Fig. 3b,c). Notably, ethanol self-administration 48 h after oxa treatment and aversion learning was accompanied by a robust increase in the power of 1–3 Hz glutamate oscillations surrounding reward-lever presses during quinine-free session (F(1, 98) = 70.03, p < 0.0001) (Fig. 3d,e). No treatment-related differences in glutamate dynamics were observed during any other session. Together, these findings indicate that oxa-noribogaine, despite inducing a global desynchronization of mPFC glutamate activity acutely, subsequently enhances synchronous low-frequency (1–3 Hz) glutamatergic oscillations during ethanol self-administration. This shift toward coordinated low-frequency activity may reflect concerted inhibitory control of the mPFC on subsequent alcohol self-administration.

## Oxa-noribogaine is also effective in alcohol dependent rats

**Figure 4:**
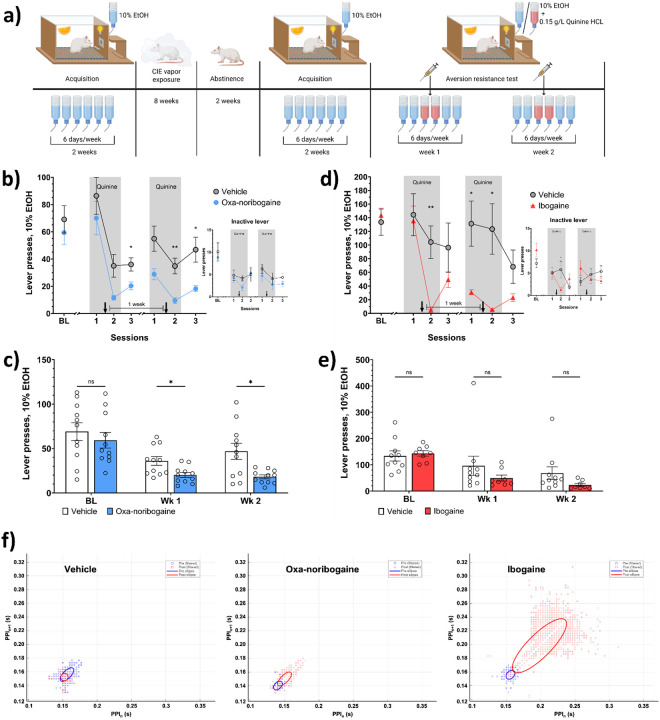
Oxa-noribogaine reduces compulsive-like alcohol self-administration in postdependent rats and does not induce cardiac rhythm deviations. a) Experiment design; initial acquisition of alcohol intake is followed by chronic intermittent ethanol (CIE) vapor exposure and abstinence (breathing normal air), before another acquisition phase and aversion resistance test similar to the design in Fig.1. b) Oxa-noribogaine (n = 11) reduces compulsive-like and quinine-free alcohol self-administration in postdependent rats; c) Consumption of quinine-free alcohol is reduced in oxa-noribogaine group after aversion resistance test on both weeks; d), e) Ibogaine (n = 8) strongly reduces compulsive-like alcohol self-administration but does not affect quinine-free intake on either week of testing. f) Representative Poincare plots of rats treated with vehicle (left), oxa-noribogaine (middle) and ibogaine (right); each individual beat-to-beat, or point-to-point interval (PPI n), is plotted against the next one (PPI n+1); measurements for baseline (blue) and post-injection (red) phases are shown. Statistical tests used: two-way repeated measures ANOVA, post-hoc Sidak’s multiple comparisons test (b-e). *P<0.05, **P<0.01.

To assess translational relevance, we tested oxa-noribogaine in a well-established rat model of alcohol dependence using chronic intermittent ethanol (CIE) vapor exposure. This paradigm induces an addiction-like phenotype characterized by escalated intake, withdrawal symptoms, and persistent alcohol seeking^31^. Rats were exposed to repeated intoxication–withdrawal cycles – which effectively produces blood alcohol concentrations (200–350 mg/dL) consistent with human intoxication^31^ – over 8 weeks, followed by abstinence and re-testing in the aversion-paired alcohol self-administration paradigm (Fig. 4a). A parallel cohort received ibogaine hydrochloride (40 mg/kg followed 3 hours later by 20 mg/kg) to directly compare efficacy.

In contrast to non-dependent animals, alcohol-dependent rats failed to reduce intake upon first exposure to quinine-adulterated alcohol, indicating compulsive-like drinking behaviour – one of the core diagnostic features of AUD in DSM-5 and ICD-11 (Fig. 4b,d). Despite this entrenched phenotype, both oxa-noribogaine and ibogaine reduced alcohol responding during quinine challenge, with a significant effect observed for oxa-noribogaine (ibogaine: F(1, 16) = 3.473, p = 0.08; oxa-noribogaine: F(1, 20) = 6.385, p = 0.02). Both oxa-noribogaine and ibogaine produced sustained suppression of alcohol self-administration after quinine removal, with reduced intake persisting across subsequent testing weeks; however, only oxa-noribogaine's effect was statistically significant (week 1: t = 2.781, df = 15.93, p = 0.0397, Hedges’ g = 1.14, 95% CI: 0.24–2.04; week 2: t = 3.043, df = 11.23, p = 0.0325, Hedges’ g = 1.25, 95% CI: 0.33–2.16; Fig. 4c). Ibogaine-treated rats did not show a statistically significant long-term reduction in drinking (F(1, 16) = 0.9086, p = 0.3541, Hedges’ g = 0.51, 95% CI: −0.44–1.45; Fig. 4e).

To determine whether aversion learning was required for durable efficacy, we administered oxa-noribogaine between baseline sessions without quinine exposure. Under these conditions, only a transient nonsignificant reduction in alcohol intake was observed (F(1, 20) = 4.037, p = 0.0582; Extended data Fig. 2c), indicating that pairing pharmacological intervention with aversive learning is necessary for significant persistent behavioural change.

Although ibogaine reduced alcohol responding during quinine challenge, this effect was accompanied by decreased inactive lever presses and observable motor impairment (t = 0.059, df = 14.11, p = 0.04), consistent with known side effects^32^. In contrast, oxa-noribogaine did not affect inactive lever responding and achieved comparable or greater suppression of alcohol intake without signs of motor dysfunction during the test sessions.

Together, these findings demonstrate that oxa-noribogaine restores behavioural control over compulsive alcohol consumption in dependent animals. Its sustained efficacy, dependence on aversion-based learning, and improved safety profile relative to ibogaine support its potential as a clinically viable therapeutic candidate for AUD. A major limitation hindering the clinical use of ibogaine is its well-documented proarrhythmic liability, which has contributed to multiple reported fatalities^10^. Although Havel et al.^11^ originally described an absence of proarrhythmic effects for oxa-noribogaine in primary human cardiomyocytes, direct *in vivo* comparison of the two compounds has been lacking. We therefore assessed their effects on cardiovascular physiology in rats using non-invasive pulse oximetry.

## Ibogaine, but not oxa-noribogaine, induces pronounced cardiac arrhythmia in rats

To directly compare cardiovascular safety, rats were monitored using pulse oximetry and rectal temperature probes before and after administration of vehicle, oxa-noribogaine (30 mg/kg), or ibogaine (40 mg/kg). Physiological parameters were recorded for 120 min following injection and normalized to individual baselines.

Beat-to-beat, or point-to-point (P–P) interval variability was assessed using Poincaré analysis to quantify short- (SD1) and long-term (SD2) rhythm variability^33,34^. Ibogaine, but not oxa-noribogaine, caused marked and significant increases in both SD1 (F(2, 68) = 19.85, p < 0.0001) and SD2 (F(2, 68) = 22.58, p < 0.0001) (Extended data Fig. 6a-b), accompanied by a distorted, non-elliptical Poincaré distribution indicative of arrhythmicity (Fig. 4f). Both compounds reduced heart rate relative to vehicle (p < 0.0001), but the bradycardic effect was substantially greater with ibogaine. Oxa-noribogaine produced a moderate reduction in heart rate without evidence of rhythm instability. Respiratory rate was reduced by oxa-noribogaine but not by ibogaine, whereas neither compound significantly altered core temperature or pulse distension (Extended data Fig. 6c-h).

These findings provide the first *in vivo* evidence that oxa-noribogaine does not produce the severe arrhythmogenic effects associated with ibogaine. This supports and extends earlier observations made in human primary cardiomyocytes^11^ and further justifies the clinical development of oxa-noribogaine for AUD.

## Oxa-noribogaine normalizes elevated BDNF and GDNF levels in alcohol-dependent rats

Ibogaine has previously been shown to reduce alcohol self-administration in rats through upregulation of GDNF expression in the VTA, alongside increases in BDNF within the mPFC and nucleus accumbens (NAcc)^21,35^. Oxa-noribogaine has similarly been reported to elevate GDNF protein levels in the mPFC and VTA five days after administration^11^. The importance of GDNF in AUD is further supported by effective GDNF gene therapy for AUD in non-human primates^36^. In humans with AUD, as well as in animal models, neurotrophin levels are typically reduced; however, compensatory increases can occur depending on the brain region, withdrawal duration, and exposure history^37–39^.

Here, animals underwent chronic intermittent ethanol exposure and 14 days of abstinence before receiving oxa-noribogaine. Brain tissue was collected five days later for protein quantification in regions implicated in compulsive alcohol use, including mPFC, anterior insula (aIns), nucleus accumbens (NAcc), and VTA using sandwich ELISA (Extended data Fig. 7a).

Alcohol-dependent rats exhibited elevated BDNF levels in both mPFC (F(2, 21) = 6.523, p = 0.0063) and aIns (F(2, 21) = 13.61, p = 0.0002) compared to non-dependent controls (Extended data Fig. 7b,c). Treatment with oxa-noribogaine normalized BDNF concentrations in both regions, bringing them to levels indistinguishable from controls (mPFC: p = 0.7385; aIns: p = 0.262). Similarly, GDNF levels in the NAcc were increased in rats (F(2, 21) = 4.056, p = 0.0324) following dependence and were restored to baseline by oxa-noribogaine (Extended data Fig. 7d,e). No significant differences were detected in the VTA (F(2, 21) = 2.127, p = 0.1441). Overall, protracted abstinence after chronic ethanol exposure was associated with aberrant elevations of BDNF and GDNF within cortico-striatal regions linked to compulsive alcohol seeking. Oxa-noribogaine reversed these maladaptive neurotrophic alterations, suggesting that its sustained behavioural efficacy may involve normalization of addiction-related plasticity rather than simple acute receptor-level effects.

## Oxa-noribogaine reduces compulsive-like drinking and prevents relapse in the ADE model using Wistar and heterogeneous stock rats

To further strengthen translational confidence and increase the likelihood of successful clinical development, it is essential to evaluate novel treatments across multiple animal models, genetic backgrounds as well as the use of female and male animals. Most preclinical AUD studies rely on inbred strains to reduce biological variability; however, this does not reflect the substantial genetic diversity found in human populations, which may influence treatment responsiveness. Therefore, we examined the effects of oxa-noribogaine in a voluntary free-choice alcohol drinking model with aversion-resistance testing (i.e., compulsive-like drinking) and a rat model for alcohol relapse in both inbred Wistar and genetically heterogeneous NIH stock rats^40–42^. This strain is derived from 8 inbred rat strains and has been maintained as an outbred population for more than 70 generations. Every rat represents a unique random mosaic of the founders^43^ and these rats represent the most highly recombinant rat intercross available.

**Figure 5:**
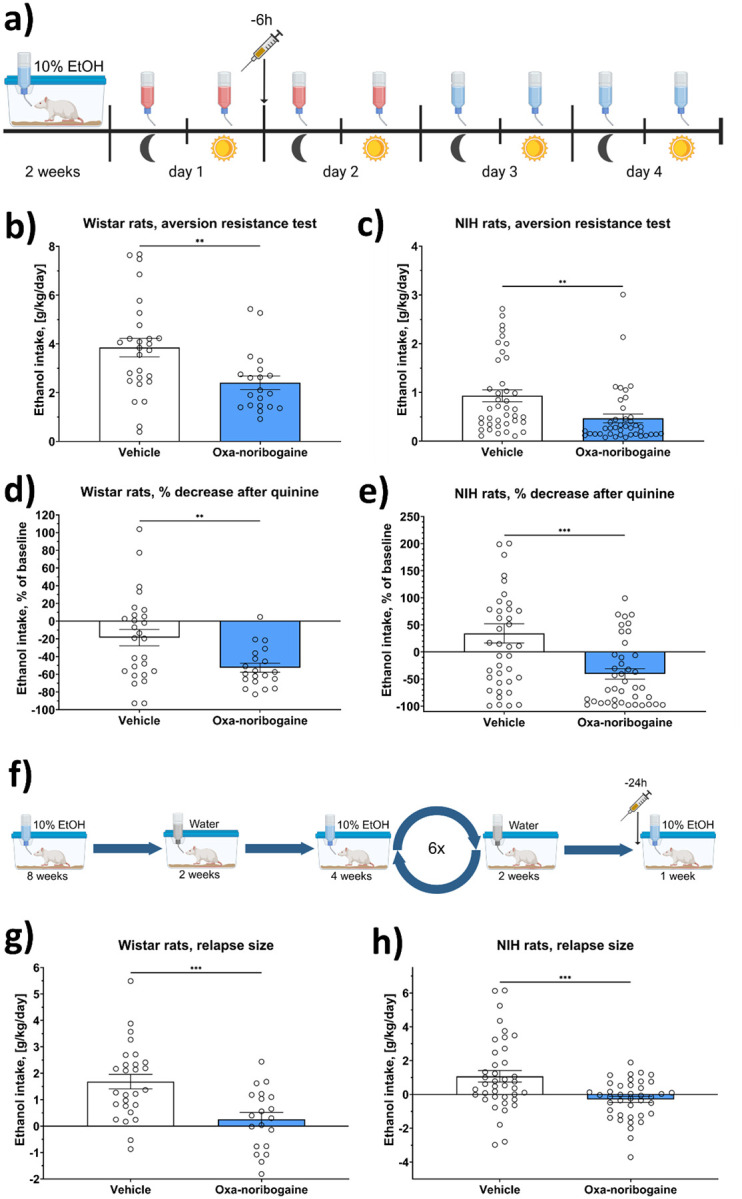
Oxa-noribogaine reduces compulsive-like alcohol drinking and prevents relapse in ADE model on ZI Wistar and NIH heterogeneous stock rats. a) Aversion resistance test with adapted protocol for home cage drinking, red bottle = 10% EtOH + 0.025 g/L quinine HCL; blue bottle = 10% EtOH; crescent = active (dark) circadian phase; sun = inactive (light) circadian phase; oxa-noribogaine was administered during the inactive phase on day 1, 6h before the start of the second active phase. b) Aversion resistance test in ZI Wistar rats, i.e. quinine-adulterated alcohol consumption 24h after injection (n = 27 per group). c) Aversion resistance test in NIH rats (n = 40 per group). d)-e) Percentage decrease in quinine-free alcohol intake 48h after treatment with oxa-noribogaine relative to baseline, f) Rat model of alcohol relapse (ADE): cycles of EtOH availability are interspersed with deprivation phases where only water is available; oxa-noribogaine was administered on the last day of deprivation, 24h before the reintroduction of alcohol bottles. g-h) Alcohol deprivation effect (ADE) is reduced in ZI Wistar (g) and NIH (h) rats after a single oxa-noribogaine injection 24h prior to restoring access to alcohol. Statistical tests used: unpaired t-test (b-h). *P<0.05, **P<0.01, ***P<0.001.

First, we assessed the impact of oxa-noribogaine on alcohol intake during quinine adulteration and a subsequent aversion-resistance phase. The design paralleled our operant experiments but was adapted to home-cage free-choice conditions. Rats had continuous access to water and 10% ethanol for two weeks. Then quinine-adulterated alcohol (0.025 g/L) was introduced at the onset of the dark phase (day 1). Quinine was removed at the end of the second dark phase (day 3). Oxa-noribogaine (30 mg/kg, i.p.) was administered 6 hours before the onset of the second drinking phase (day 2) (Fig. 5a).

As in operant testing, oxa-noribogaine significantly reduced aversion-resistant drinking in Wistar rats, evidenced by lower intake of quinine-adulterated alcohol on day 2 compared with vehicle-treated controls (t = 2.864, df = 45, p = 0.0063, Hedges’ g = 0.83, 95% CI: 0.23–1.43) (Fig. 5b). While vehicle-treated rats also showed a modest reduction in quinine-free alcohol drinking (mean = −18.7%, SEM = 9.3), oxa-noribogaine–treated rats displayed a substantially greater decrease (mean = −52.6%, SEM = 5.0), which was significantly larger than the vehicle group (t = 2.91, df = 45, p = 0.0056, Hedges’ g = 0.84, 95% CI: 0.24–1.45) (Fig. 5d).

We next evaluated oxa-noribogaine’s efficacy in relapse-like drinking using the alcohol deprivation effect (ADE) model^44,45^. Single-housed rats received continuous access to water and 10% ethanol initially for 8 weeks, followed by a 2-week deprivation period. Drinking phase was then shortened to 4 weeks, and this cycle of drinking and deprivation was repeated six times over 10 months. On the final day of the sixth deprivation phase, rats received oxa-noribogaine (30 mg/kg, i.p.) 24 hours before alcohol reintroduction, and intake was monitored for one week (Fig. 5f).

Vehicle-treated rats exhibited a robust ADE, increasing their alcohol consumption by 143% (SEM = 8.3) on the first day of renewed access, resulting in an average relapse size of 1.8 g/kg (SEM = 0.3). In contrast, oxa-noribogaine greatly reduced relapse size to 0.3 g/kg (SEM = 0.36), corresponding to a significantly smaller intake increase of 109% (SEM = 5.60) (t = 3.68, df = 45, p = 0.0006, Hedges’ g = 1.07, 95% CI: 0.45–1.68) (Fig. 5g). These findings demonstrate that oxa-noribogaine lowers alcohol consumption consistently across both operant and voluntary paradigms as well as reducing alcohol relapse.

To further assess generalizability across diverse genetic backgrounds, we repeated the quinine aversion and ADE experiments in NIH heterogeneous stock rats as part of a multi-site validation effort^42^. In the aversion-resistance test, oxa-noribogaine significantly decreased quinine-adulterated alcohol intake (t = 3.076, df = 78, p = 0.0029, Hedges’ g = 0.68, 95% CI: 0.23–1.13), with the effect persisting through days 3 and 4 after quinine removal (t = 3.716, df = 78, p = 0.0004, Hedges’ g = 0.82, 95% CI: 0.37–1.28) (Fig. 5c,e). Oxa-noribogaine also completely abolished the ADE in both male and female NIH rats (t = 3.586, df = 78, p = 0.0006, Hedges’ g = 0.79, 95% CI: 0.34–1.25) (Fig. 5h).

Together, these results confirm that oxa-noribogaine reliably reduces compulsive-like drinking and prevents relapse across multiple behavioural paradigms, environmental conditions, in both sex and genetic backgrounds. These findings, consistent with effects observed in the CIE model, provide strong translational support for advancing oxa-noribogaine as a therapeutic candidate for AUD.

## Discussion

Ibogaine has attracted renewed interest for its capacity to alleviate withdrawal and reduce substance use across multiple addictions, yet its clinical utility is limited by cardiac arrhythmia risk linked to hERG channel blockade. Oxa-noribogaine was developed to retain ibogaine’s anti-addictive pharmacology while minimizing toxicity. Here we show that oxa-noribogaine reduces compulsive alcohol intake in both non-dependent and alcohol-dependent rats, lacks arrhythmogenic liability *in vivo*, and exerts its behavioural effects through context-dependent enhancement of aversion learning accompanied by dynamic reorganisation of mPFC glutamatergic activity.

A central conceptual advance of this study is the introduction of an aversive alcohol-paired learning paradigm as a translational analogue of the psychedelic “setting.” In clinical contexts, psychedelic therapy combines pharmacologically induced neuroplasticity with emotionally salient experiences that allow patients to confront maladaptive behavioural patterns. In our model, quinine-adulterated alcohol serves as a controlled aversive context that challenges habitual alcohol seeking. Oxa-noribogaine did not simply suppress drinking acutely; rather, it strengthened the impact of aversive feedback, producing sustained reductions in alcohol intake that persisted after quinine removal. When administered without aversive pairing, its effects were transient, indicating that pharmacology alone is insufficient for durable behavioural change. Thus, oxa-noribogaine appears to open a window during which negative outcomes are more effectively encoded into future decision-making, conceptually similar to how psychedelics have been shown to reopen critical periods for adaptive learning in the social reward domain, as recently reported^46^.

Mechanistically, this effect is consistent with transient NMDA receptor antagonism combined with partial KOR engagement. Comparative pharmacology showed that NMDA antagonism recapitulates acute suppression of aversion-paired drinking, whereas KOR blockade attenuates oxa-noribogaine’s effect, suggesting that neither pathway alone accounts for its persistence. The durable behavioural outcome likely reflects its multi-faceted “matrix pharmacology”^26^, in which coordinated modulation of glutamatergic and opioid systems produces circuit-level reorganisation.

*In vivo* fiber photometry revealed a two-phase cortical response. Oxa-noribogaine acutely desynchronized mPFC glutamatergic activity, increasing entropy and shifting spectral organisation without altering total glutamate levels. During subsequent aversion-paired sessions, however, low-frequency (1–3 Hz) glutamatergic oscillations became selectively enhanced and time-locked to alcohol-seeking behaviour. This transition from transient desynchronization to coordinated low-frequency activity suggests a reset-and-reorganise mechanism: temporary disruption of maladaptive cortical synchrony followed by strengthened, behaviourally relevant prefrontal control. Such dynamics parallel observations with other rapid-acting NMDA antagonists, where brief inhibition facilitates subsequent synaptic remodelling and cognitive flexibility^47,48^. Neurotrophic signalling further supports this model. Alcohol-dependent rats in protracted abstinence exhibited elevated BDNF and GDNF levels in cortico-striatal regions implicated in compulsive alcohol seeking. Rather than further increasing neurotrophins, oxa-noribogaine normalized these elevations to control levels. Although neurotrophins are often interpreted as protective, excessive or dysregulated BDNF/GDNF signalling may reflect maladaptive hyperglutamatergic drive during early abstinence^49,50^. By transiently attenuating NMDA-dependent excitation, oxa-noribogaine may recalibrate neurotrophic signalling toward a balanced state that favors adaptive plasticity and stabilizes cortical–striatal communication.

Importantly, oxa-noribogaine produced similar or stronger and more sustained reductions in alcohol drinking than ibogaine, without inducing cardiac arrhythmia or motor impairment. This improved safety profile, combined with efficacy in dependent animals and across heterogeneous genetic backgrounds, strengthens its translational potential. Together, our findings position oxa-noribogaine as a promising therapeutic candidate for alcohol use disorder that operates at the intersection of pharmacology and context. By transiently destabilizing maladaptive glutamatergic states and enhancing the salience of aversive feedback, it promotes durable behavioural recalibration. More broadly, our aversion-paired learning paradigm provides a framework for modelling the interaction between neuroplastic drugs and psychologically meaningful context, advancing the preclinical study of psychedelic-assisted therapies beyond receptor pharmacology alone.

## Materials and Methods

### Animals.

For all experiments involving operant alcohol self-administration, chronic intermittent ethanol vapor exposure, fiber photometry, ELISA, MALDI-MS and pulse oximetry, male Wistar rats (Charles River, Sulzfeld, Germany, id: Crl:WI(Han)) at an age of 7 weeks were used at the start of each experiment. Upon arrival at the facility, rats were housed in eurostandard polycarbonate Type IV cages (480 × 375 × 210 mm, Tecniplast, Italy) in groups of 4 animals per cage under reversed light cycle (lights OFF 7:00, lights ON 19:00) in a temperature (22 °C±1 °C)- and humidity (40%±5%)- controlled room. Cages were filled with 2–3 mm bedding made of stem-sterilized aspen wood (Abedd, Latvia) and bricks of gnawing wood were provided for the purpose of simple enrichment. Throughout the entire duration of each experiment, rats received 22 g of standard chow food (Altromin, Germany) per day; tap water was provided ad libitum.

For the home cage drinking experiments in the ADE model, one batch of selectively bred male Wistar rats (“Crl:WI(Han)”, (RS:0001833)) with robust alcohol drinking and ADE phenotype and a batch of 40 male and 40 female NIH-HS (heterogeneous stock) rats at the age of 5 weeks were used. These rats were single-housed in Type III cages (425 × 266 × 185 mm, Tecniplast, Italy) with the same type of bedding but without any additional enrichment. Before the start of experiments, rats were let to habituate to the room conditions for one week. Cages and water bottles were changed once per week, and food was provided ad libitum All experiments were approved by the institutional Committees on Animal Care and Use, by the respective German (Berlin, Nurnberg, Karlsruhe) and Italian authorities and were performed in accordance with the European guidelines.

### Reagents.

Ibogaine-HCl was obtained from a commercial vendor (Coryn Pharmaceuticals LLC), noribogaine-D4 was synthesized as shown in Supplemental Information, oxa-noribogaine-HCl were synthetized as described in Havel et al.^11^ Memantine (Merck, Germany), U-50488 (Merck, Germany) and Aticaprant (MedChemExpress, Sweden) were sourced from local European suppliers. Water for injections (Ampuwa, Fresenius Kabi, Germany) was used as a vehicle to prepare all drug solutions. For each intraperitoneal and subcutaneous injection, the total volume of injection was set at 2 mL/kg. Thus, to achieve e.g. 30 mg/kg dose, a solution of 15 mg/mL was prepared and injected into each animal according to its body weight. All solutions were freshly prepared from water for injections with addition of solubilising agent Tween-80 and/or non-polar solvent DMSO, when applicable, as indicated in the table below. Ready solutions were allowed to cool to room temperature before injections were performed.

**Table:** 

Compound	Dose (rat)	Concentration (solution)	Tween-80	DMSO	Water bath
Oxa-noribogaine	30 mg/kg	15 mg/mL	1%	–	60 °C, 5 min
Ibogaine	40 mg/kg	20 mg/mL	5%	0.5%	60 °C, 5 min
Memantine	25 mg/kg	12.5 mg/mL	–	–	–
U-50488	10 mg/kg	5 mg/mL	–	–	–
Aticaprant	1 mg/kg	0.5 mg/mL	5%	1%	60 °C, 5 min

### Operant alcohol self-administration.

After one week of habituation to the housing room conditions, rats underwent operant training in 12 alcohol self-administration sessions. Training took place during the animals’ active (dark) phase of the light cycle; each session lasted for 30 minutes and was done every day except Sundays, i.e. 6 days/week. To signal the reward availability, 5 drops of orange oil (Manske GmbH, Germany) were put onto the metal tray in each operant box (environmental cue). Upon the session start, two levers from opposite sides of the operant box would extend and pressing on the left lever (FR1) would result in delivery of 40 μL 10% ethanol into the left reward receptacle. Reward delivery was accompanied by 3 seconds of blinking white light on the same (left) side and a timeout of 5 seconds, during which presses on the left lever were recorded by no additional reward was delivered. Presses on the right (inactive) lever produced identical mechanical noises as the reward lever, were recorded but did not deliver any liquid, thus serving as control measure for locomotor activity and to confirm the acquisition of alcohol intake. A total of 12 sessions was done to achieve a 10:1 ratio of reward vs. inactive lever responses in all animals, after which the main experimental part began (Extended Data Fig.2b). Rats were randomized into two groups by the amount of average reward lever presses during the last 3 days of training with nearly identical means (95.7, 96.4) and standard deviations (45.5, 46.1) between them. On the days 3 and 4 of the first week of experiment (after the initial acquisition training), all groups would receive 0.15 g/L quinine hydrochloride added to the 10% ethanol solution. This dose was selected based on previous experiments in our laboratory with this rat strain and identified as the most reliable in terms of producing aversion to ethanol and thus hindering its rewarding properties for animals (Extended Data Fig.2a). On days 5 and 6, quinine was removed from the solutions and rats continued to receive normal 10% ethanol solution. The following week this schedule was repeated again, with two days of quinine-adulterated alcohol in the middle preceded and followed by days of normal alcohol availability. Oxa-noribogaine was administered on days 3 and 9, first dose (30 mg/kg) two hours and the second dose (20 mg/kg) five hours after the end of the training session on that day (Fig. 1 a). The two-hour administration point was chosen based on previous experiments by T. Buchborn et al.^51^ as effective in producing learning-related therapeutic psychedelic effects. Two doses 3 hours apart were chosen after discussion with D. Sames in order to extend the time of exposure to psychoactive effects and to bring the pharmacokinetic profile of iboga compounds in rats closer to human condition, where ibogaine experience can last for up to 18–36 hours.

### CIE vapor exposure.

After completing the acquisition phase of alcohol self-administration (12 sessions), rats in their home cages were placed into chambers with constant air flow of 14 L/min, with 16 h/day ethanol vapor supply through an automated system which consisted of a rotary pump connected to 96% ethanol canister and a custom-built heating (vaporization) device. Chambers were equipped with airtight locking door and air in-/outlet system to ensure balanced exposure to ethanol vapor with proper air circulation. The exposure began at 1:00 and stopped at 15:00, covering the entire active (dark) phase of the animals' circadian cycle (lights ON 15:00, lights OFF 3:00). A separate cohort of rats was kept in the same room outside of vapor chambers (vapor-naïve rats, controls). Twice per week, right after the end of that day's vapor exposure cycle, tail blood samples of ~10 μL were taken and analyzed for blood alcohol levels (BALs) using the Analox AM1 system (Analox Instruments, UK). The rotation speed of the rotary pump supplying 96% alcohol into the heating system was adjusted based on the results of these measurements to ensure BALs remain in the range of 200–350 mg/dL. In total, rats were subjected to 8 weeks of intermittent cycles of 16h/day ethanol vapor and 8 h/day clean air inhalation, effectively mimicking binge-deprivation dynamics of AUD development. At the end of CIE exposure, ethanol vapor supply was stopped and rats underwent 2 weeks of abstinence while breathing clean air. For the ELISA experiment, rats were treated with vehicle or oxa-noribogaine on day 14 of abstinence and sacrificed 5 days later (see “ELISA” section). For operant experiments with ibogaine and oxa-noribogaine, a second acquisition phase was repeated after 14 days of abstinence before proceeding with aversion resistance tests and drug administration (Fig. 5 a).

### Stereotactic surgery.

Surgeries were performed based on the standard protocol by Cetin et al.^52^ with some in-house modifications. Induction of anesthesia was performed with 4.5% isoflurane (Baxter GmbH, Germany) and step-wise lowered to 2% and maintained throughout the surgery. A subcutaneous bolus of 5 mg/mL Carprofen (Rimadyl, Zoetis, Germany) was injected to provide additional pain relief and the eyes were covered with eye cream (Bepanthen, Bayer, Germany). Once the rat was deeply anesthetized, the skull between the ears was shaved with an electric hair trimmer (Aesculap Schermachinen, Germany), disinfected with iodine (Iodovet-spray, CP-Pharma, Germany) and cleaned from residual hair with alcohol swab (Teqler, Lotus NL B.V., Netherlands). Topical application of 1% lidocaine (Xylocain, Aspen Pharma Trading Limited, Ireland) was performed on the head surface and inside ear cavities to ensure painless incision and ear bar placement. Rats were fixed in a stereotactic apparatus (Model 902, David Kopf Instruments, USA) and placed on a heating pad with 39°C (2 degrees higher to account for heat dissipation) to maintain body temperature. Upon incision, excessive blood was cleaned off with sterile cotton swabs and a surgical Alm retractor (AgnThos, Sweden) was inserted. Connective tissue was completely removed from the skull surface, and any sipping blood was quickly removed and identified bleeding spots were cauterized (Bovie Medical Corporation, USA). The position of tilt and scaling of the skull was corrected and the skull was pretreated with Super-Bond Red Activator (Sun Medical, Japan) for 10 minutes. Coordinates for craniotomy were adjusted based on the animal body weight on the day of surgery using the excel macros from Yang et al.^53^ For the mPFC area the following coordinates were used^54^: AP 3.2, ML ±0.6, DV −3.45. Craniotomy was done manually by using 0.6 mm dental drill bits (Emil Lange Zahnbohrerfabrik, Germany); approximately 1 mm away from the desired craniotomy position, an indentation was made in the skull using the same drill bit and an 0.8-mm-screw was inserted in it to be used as an anchor for the fiber optic cannula. Rats were unilaterally injected with 600 nL of iGluSnFR virus (pAAV.hSynapsin.SF-iGluSnFR.A184S); infusions were performed using a microinjection syringe (NanoFil, World Precision Instruments, USA) mounted on a microinjection pump (UMP3, World Precision Instruments, USA). To reduce tissue damage the syringe was slowly lowered down to the level of the target region (~0.5 mm/min). The virus solution was infused at a rate of 200 nL/min and the syringe was left in place for 10 min before retracting. Zirconia optic fiber cannulas with a fiber diameter of ⌀ 400 μm (5 mm length) and ferrule diameter of 2.5 mm (Doric Lenses Mono Fiber-optic Cannula) were implanted 200 μm above the infusion site. The length of the implanted fiber was 0.4–1.5 mm longer than needed to reach the depth of the target implantation site. To reduce tissue damage the fiber implant was slowly lowered to the level of the target coordinates (~0.5 mm/min). The implant was fixed to a screw by covering the skull surface with dental adhesive (Super-Bond, Sun Medical, Japan) spread across the exposed skull only sparing the outer edges, and an additional layer of protection with dental cement (Ortho-Jet Black, Lang Dental Mfg.Co., USA) was added after 8 minutes of curing time to create a dome shape, further embedding the implant. Once the dental cement had finished solidifying for 8 minutes, the Alm retractor was removed and the skin was sutured using absorbable suture (Marlin violet) leaving only the top of the cannula exposed. At regular intervals throughout the surgery, saline was injected subcutaneously to the total volume of 5 mL to keep the rats hydrated. After the surgery, the animal was kept in an isolation cage on a heating plate for 20 min until full awakening. For the following two days the animal was single-caged and received further analgesia via s.c. injection of 5 mg/kg carprofen and potential post-surgical pain was assessed using the rat-grimace-scale. After two days of recovery in a single cage, rats were transferred back to their cage mates; a period of 4 weeks was given to allow for full expression of the glutamate sensor.

### Fiber photometry (wired).

Fiber photometry acquisition was performed with a Doric fiber photometry system (Doric Neuroscience Studio, version 5.4.1.23). For iGluSnFR, an excitation wavelength of 470 nm was used for the glutamate dependent signal while 405 nm functioned as a “pseudo-isosbestic” signal. Recordings were done at 20 Hz with an exposure time of 21 ms. The gain was individually adjusted for every recording to bring the signal to ~30’000–40’000 range. Before the start of each recording, the fiber optic cable was bleached at 5x light intensity at 20 Hz for 30 minutes to reduce autofluorescence. The cable was then attached to the implanted ferrule using a copper sleeve, and the animal was transferred to a transparent recording box (22 × 38 × 28 cm). The animal was able to freely walk inside the box as the fiber optic cable was attached to a rotary joint that would automatically follow the rotation of the cable and reduce tension. At 90 min, the animal was gently taken out from the box while still being attached to the fiber optic cable, injected intraperitoneally with vehicle (n = 3) or oxa-noribogaine (n = 3, 30 mg/kg), and placed back into the recording box. The recording continued for an additional 90 minutes, reaching a total of 3 hours (180 min).

The fiber photometry signal was processed using a custom MATLAB pipeline. The first step of preprocessing was detrending of the raw photometry signal to remove artifacts caused by gradual signal decay or rise resulting from photobleaching and temperature fluctuations. To that end, a 3rd order polynomial function was fitted to the dependent and independent signal and subtracted from them. Additionally, a high-pass Butterworth filter with a threshold of 0.2 Hz was applied to both signals to correct for transient artifacts. After detrending, the dependent and isosbestic signals were standardized by the standard deviation of the isosbestic signal. That way, the arbitrary fluorescence intensity unit of the signals is transformed into a z-score which is centred around 0. Detrended and normalized signal was then Fourier-transformed and analyzed in the 0.2–4 Hz frequency band. The amplitude spectral density (ASD) was computed in sliding windows, and the area under the ASD curve (AUC) was used as a frequency-domain measure of signal power. In parallel, the root mean square (RMS) amplitude of the band-pass filtered signal (0.2–4 Hz) was calculated as a time-domain measure of overall activity strength. Both parameters were normalized to the pre-drug baseline (z-score) for comparison.

### Wireless fiber photometry.

Male Wistar rats (Charles River, Germany) at 9 weeks age were used in this experiment. Rats underwent stereotactic surgeries and were implanted with fiber optic cannulas into the mPFC above the expression site of iGluSnFR sensor (see the protocol above). One month after the surgery, rats began operant training according to our protocol (see “operant self-administration”). In order to habituate the rats to carrying the wireless photometry device on their head during the operant session, each training began by gently attaching a mock (dummy) headstage (Tele FiPho, Bio Research Center Co., Japan) to the fiber optic cannula before placing the animal into the chamber. The dummy headstage had identical size and weight (~2 g) to the actual wireless transmitter. In total, rats underwent 14 operant sessions with 10% EtOH during the acquisition phase. Starting from day 15, each session was conducted with a real photometry transmitter headstage. The headstage was gently attached to the fiber optic cannula and fixed with two screws to prevent it from detaching; the headstage was then turned on and recording started immediately using the original TeleFipho software (excitation wavelength peaked at 470 nm with a 445–490 nm filter band; emission wavelength with a 500–550 nm filter band, sampling frequency of 100 Hz). The first 5 minutes of recording were used to adjust the offset and light power to bring the signal to ~30’000–40’000 range and were discarded from analysis. The Med-PC script was modified to record timestamps of each lever press (rewarded and non-rewarded) and to send TTL pulse at the moment of the session’s start and finish for later alignment with photometry signal during analysis. Because of the small sample size and to allow for within-subject comparison of glutamate activity under vehicle or oxa-noribogaine, all animals received vehicle injection during the first week of aversion resistance test, while oxa-noribogaine was administered during the second week. All other procedures remained the same as in other operant experiments.

Analysis was performed using custom MATLAB script. Detrended and filtered glutamate signal was transformed into z-score and used for further analysis. Timestamps of recorded events were aligned with the photometry signal and a 3-second window surrounding the reward lever press was extracted and averaged between individual animals for each day. For each individual animal, glutamate signal power (dB) was compared between vehicle and oxa-noribogaine treatment on baseline, both days of quinine-adulterated alcohol and the first day of quinine-free alcohol self-administration sessions. Data were analyzed using repeated-measures two-way ANOVA with statistical significance set at p < 0.05. Statistical analyses were performed using GraphPad Prism 10 (GraphPad Software, San Diego, CA).

### Pulse oximetry.

Male Wistar rats (Charles River, Germany) at the age of 9 weeks (n = 9) were used in this experiment. A rat was placed into anesthesia induction chamber (EZ Systems, USA) with isoflurane supplied at 4% with 1.0 L/min air flow rate. After 4 minutes, the rat was quickly removed from the chamber and thoroughly shaved around the neck area (~2 cm wide ring) by using the electric trimmer (Aesculap Schermachinen, Germany) to allow for appropriate placement of the pulse oximetry sensor. The rat was then placed back into the induction chamber for additional 3 minutes, and the rate of isoflurane was reduced to 3.5%. After that, the air flow rate was reduced to 0.8 L/min, isoflurane supply switched from the induction chamber to the breather, and the rat was placed on the heating pad (EZ Systems, USA) set to 39 °C with its nose fixed firmly in the breather cone. To check for the absence of reflexes, the rat’s rear paw was squeezed with forceps in between fingers. Eyes were covered with Bepanthene protective cream (Bayer, Germany) and the tip of the rectal temperature probe was covered with it before insertion into the animal. Pulse oximetry sensor (MouseOx large collar, Starr Life Sciences Corp., USA) was placed around the neck right above the carotid artery on the right side. The recording began shortly after the placement of the sensor and the total preparation time did not exceed 10 minutes. 5 minutes after the start of the recording, isoflurane supply was reduced to 2.5 %; at 25 minutes – further down to 2%, and remained at this rate until the end of measurement. At 55 minutes, the rat was subcutaneously injected with vehicle, oxa-noribogaine (30 mg/kg) or ibogaine (40 mg/kg) (n = 3 per group) and the recording continued until 150 min. In total, each animal spent 10 minutes under deep anesthesia (3.5%–4% isoflurane) during the preparation phase, which was progressively decreased to 2% throughout the next 25 minutes and kept at that rate until the end of measurement. Sensor parameters (heart rate, breath rate, core temperature, pulse distension) recorded during the time period between 25 and 55 minutes (30 minutes at 2% isoflurane) were taken as baseline and compared with post-injection period (1 hour 30 minutes at 2% isoflurane). Data were analyzed using a custom MATLAB script with each parameter processed as time series; sliding window analysis was applied with 100 s window size and 10 s step, centred around the time of injection (t0). Each window raw value was transformed into z-score relative to mean and SD of baseline period (values before t0). After averaging transformed z-scores of post-injection periods for each treatment group, these values were compared between groups using one-way ANOVA.

To detect cardiac arrhythmia, raw heart rate signal was analyzed by using the Poincare plot, in which each P–P interval (time between consecutive heartbeats) is plotted against the following interval. The resulting scatter distribution was quantified by fitting an ellipse whose short (SD1) and long (SD2) axes represent short-term and long-term heart rate variability, respectively. Distortions of the elliptic shape of PPI clustering and an increase in SD1 reflects greater beat-to-beat irregularity and thus serves as a proxy for arrhythmic activity^33,34^.

### MALDI-MSI experiment.

#### Chemicals

1,5-diaminonaphthalene (1,5 - DAN), hydrochloric acid, Mayer's hemalaun solution, eosin Y-solution 0.5%, hydrochloric acid, xylene, and Eukitt were sourced from Merck KGaA (Darmstadt, Germany). L-Tryptophan-(indol-d5)(Trp-D5), Acetonitrile (ACN), LC-MS grade water, methanol (MeOH) and ethanol (EtOH) were purchased from VWR Chemicals (Darmstadt, Germany). ESI-L low concentration tuning mix for calibrating MALDI-MSI measurements was acquired from Agilent Technologies (Waldbronn, Germany). Conductive indium tin oxide (ITO)-coated glass slides were sourced from Bruker Daltonics (Bremen, Germany).

#### Tissue preparation

Rat brain tissue was equilibrated at −20°C in a cryostat (Leica Biosystems, Nussloch, Germany) for 4h. Using a sterile scalpel, the rat brains were carefully cut in their two brain hemispheres. The Left Hemisphere was further cut at Interaural 3.70 mm and Bregma −5.30 mm. The anterior part of the left hemisphere was then mounted onto 2% carboxymethylcellulose (CMC), and 10 μm sections of each brain were acquired aiming at a section plane between Interaural 10.60–10.70 mm and Bregma 1.60–1.70 mm. Sections were thaw-mounted onto ITO-coated glass slides (Bruker). One biological replicate each of the Control and Oxa groups was sectioned onto a single ITO slide, resulting in three slides per group to reduce slide-to-slide variation^55^. In order to analyze each brain using FMP-10 on-tissue chemical derivatization and regular MALDI-MSI in negative Ion mode, an additional set of three slides was prepared using consecutive tissue sections: one set for positive ion mode with chemical derivatization, and one set for negative ion mode using 1,5-DAN matrix. The prepared slides were stored in vacuum-sealed slide mailers at −80 °C until measurement.

#### MALDI-MS Imaging sample preparation with 1,5-DAN

Slides were equilibrated at room temperature (RT) in a desiccator for 30 min. Brightfield images were acquired immediately afterward using an Aperio CS2 Slide Scanner (Leica Biosystems), followed by matrix application. 10 mg 1,5-DAN solution (10 mg/mL) was dissolved in a mix of acetonitrile (ACN) and water 60:40 (v/v) and acidified with 60 μL 6 M HCl. 120 μL Tryptophan-d5 (Trp-D5) from a 5 mg/mL stock solution was included as an internal standard with a final concentration of 0.1 mg/mL. The matrix solution was sonicated for 15 min. Matrix was deposited in eight layers onto the tissue sections using an M5 Sprayer (HTX Technologies LLC, Chapel Hill, USA). The spray parameters were as follows: nozzle height of 40 mm, CC pattern with 2 mm track spacing, temperature of 65°C, gas flow rate of 2 L/min, gas pressure of 10 psi, solution flow rate of 70 μL/min, nozzle velocity of 1200 mm/min, and a drying time of 10 seconds between layers. After matrix application, the slides were immediately transferred to the mass spectrometer for analysis.

#### MALDI-MS imaging after on-tissue chemical derivatization using FMP10

For the preparation of coronal rat brain sections, deuterated internal standard was applied before application of the derivatization matrix. A solution of 3.2 μM noribogaine-D4 (NB-D4) and 1.6 μM Psilocin-D10 (PN-D10) was prepared in methanol/water (70:30 (v/v)) and sprayed onto the sections using a syringe pump connected to a M3 Sprayer (HTX Technologies LLC) to account for small volumes of deuterated standard. Nine layers of deuterated standard mix were applied with a spray nozzle height of 40 mm, CC pattern with 2 mm track spacing, temperature of 60°C, gas flow rate of 2 L/min, gas pressure of 10 psi, solution flow rate of 50 μL/min, nozzle velocity of 1200 mm/min, and a drying time of 5 seconds between layers. After application of internal standard, the slides were dried in a desiccator for 10 min followed by application of freshly prepared reactive matrix FMP-10 (tag-on, Uppsala, Sweden) 1.82 mg/mL in can/water (70:30 (v/v)). FMP-10 solution was applied onto the sections in 20 layers using a M5 Sprayer (HTX Technologies LLC). The spray parameters were as follows: nozzle height of 40 mm, CC pattern with 2 mm track spacing, temperature of 70°C, gas flow rate of 2 L/min, gas pressure of 10 psi, solution flow rate of 80 μL/min, nozzle velocity of 1100 mm/min, and a drying time of 2 sec between layers. After derivatization, the slides were immediately transferred to the mass spectrometer for analysis.

#### MALDI-MS Imaging Measurements

MALDI-MS Imaging of derivatized sections was performed at 20 μm pixel size in positive Ion mode using a timsTOF flex mass spectrometer (Bruker Daltonics) with flexImaging software 7.5 (Bruker Daltonics). The instrument was calibrated externally using ESI tune mix and internally using the FMP-10 cluster ion at m/z 555.2231 for one-point calibration of all spectra during measurement^56^. Mass spectra were collected in an m/z range of 320 – 1300 with 300 laser shots at a frequency of 10,000 Hz and a laser field size of 20 μm. Radiofrequency (RF) at Funnel 1 was set to 250 Vpp, Funnel 2 RF to 250 Vpp and the Multipole RF to 250 Vpp. The collision energy was 5 eV with a Collision RF of 1500 Vpp. The low mass was set to 410 and the pre pulse storage to 8 μs. Transfer time was set to 80 μs. MALDI-MS Imaging with 1,5-DAN was performed at 20 × 20 μm pixel size in negative Ion mode using a timsTOF flex mass spectrometer with flexImaging software 7.5. The instrument was calibrated using ESI tune mix and Trp-D5 at peak at m/z 208.1141 [M–H]^−^ as online calibrant for one-point calibration of all spectra during measurement. Mass spectra were collected in a range of m/z 50 – 1000 with 200 laser shots at 10,000 Hz and a laser field size of 20 μm. Funnel 1 RF was set to 220 Vpp, Funnel 2 RF to 200 Vpp and the Multipole RF to 200 Vpp. The collision energy was 4 eV with a Collision RF of 800 Vpp. The low mass was set to 70 and the pre pulse storage to 7 μs. Transfer time was set to 65 μs.

#### Tissue staining

Hematoxylin and eosin (H&E) staining was performed on consecutive sections using a standard protocol. Briefly, slides were stained in hematoxylin for 1 min, rinsed under tap water for 1.5 min, and briefly dipped in distilled water. This was followed by differentiation in acidic alcohol (1 min) and additional rinsing in distilled water. Sections were then incubated in the bluing solution for 2 min, rinsed, and counterstained with eosin for 1 min. After a final rinse, tissues were dehydrated in graded ethanol (80%, 96%, 100%; 1 min each) and cleared in xylene for 2 min. Coverslips were mounted with Eukitt, and brightfield images were acquired the following day using an Aperio Slide Scanner (Leica Biosystems).

#### Data Analysis and statistical analysis

All measurements were imported into SCiLS Lab software (2025a Pro Version; Bruker Daltonics) for data analysis. Data collected in negative ion mode with 1,5-DAN were normalized to the internal standard Trp-D5 at m/z 208.1141 [M–H]^−^. In this data, the mPFC was manually annotated as region of interest (ROI) in each brain section using QuPath (Version 0.5.1), based on the Rat brain atlas^54^. Annotated regions were exported as .sef files via the SCiLS extension for QuPath (QuPathToSCiLS v1.4) and co-registered with the MALDI-MSI datasets in SCiLS Lab, resulting in new region definitions for each section. ImzML files for each section and the corresponding mPFC regions were exported for further analysis. A feature list was generated using an estimated signal-to-noise ratio (SNR) threshold of 3. The imzML files were additionally uploaded to Metaspace (https://metaspace2020.org/), and annotation tables were exported as .csv files. Annotation tables and feature lists across sections were merged; feature list curation was performed in R. Only Metaspace annotations with a false discovery rate (FDR) ≤10% were included in the final feature list. Using the Cardinal R package^57^, all imzML files were then aligned to the curated feature list with a tolerance of ± 0.049 Da using an in-house R script^58^. Data collected in positive ion mode after chemical derivatization using FMP10 were normalized to the internal standard NB-D4 at m/z 569.3298 [M+FMP10]^+^. ImzML files for each section were exported and aligned to a manually curated feature list of targets using an in-house R script^58^. Further analysis of data from both measurements was done using the Cardinal package^57^ and further packages for data wrangling and management in R. Statistical testing was conducted using unpaired t-tests for the relevant m/z values, based on either mean spectra of the entire section or within the mPFC ROIs. To minimize inter-individual variability, mPFC data was normalized to the global mean signal of each section: Normalized Intensity = Mean Intensity (mPFC) / Mean Intensity (Whole brain).

### Sandwich ELISA.

A total number of 24 male Wistar rats (7 weeks old) was used. First, rats (n = 16) were subjected to 8 weeks of intermittent ethanol vapor exposure, followed by 14 days of abstinence where rats were breathing normal air. A separate group of rats (n = 8) was housed in the same room outside vapor chambers and thus served as healthy controls. On day 14, half of the rats (n = 8) exposed to CIE were injected with 30 mg/kg oxa-noribogaine, followed by a second dose of 20 mg/kg 3 hours later. After 5 days (day 19 of abstinence) rats were decapitated, brains quickly extracted, flash-frozen in isopentane and transferred to −80°C freezer for storage. After that, brains were sectioned on cryostat (Leica) at 100 μm slice thickness and sections of interest (mPFC, aIns, NAcc, VTA) were collected by using a punch cutter and transferred into pre-weighed eppendorf tubes. Approximately ~10 mg of tissue per region was collected, ranging from 5 mg in the smaller regions (e.g. VTA) to 15 mg in the larger ones (aIns). To release the neurotrophic factors from their receptors and chaperones, a RIPA buffer (R0278, Sigma-Aldrich, Germany) was used. The frozen tissue punches were suspended in 100 μl of the RIPA buffer with the addition of protease inhibitor (ThermoScientific, Germany) cocktail (10 μL inhibitor per 1 mL RIPA stock solution). The suspensions were then sonicated in short bursts at low intensity for 5 seconds each, using a Labsonic U B.Braun sonicator. The samples were then left on ice for 30 min and sonicated again, to reach homogeneity. The homogenates were then centrifuged at 14.000 rpm and 4°C for 20 min. 90 μl of clear supernatants were aspirated and then transferred into clean tubes.

Depending on the protein of interest (BDNF or GDNF), the following steps were slightly different. For BDNF, 25 μL of clear supernatant were transferred to the empty tube to which additional 25 μL RIPA buffer was added, bringing the total volume to 50 μL and a dilution of 1:2. These 50 μL were used for BCA assay in duplicate with 25 μL volume of sample per well. The remaining 65 μL of clear supernatant were mixed with 260 μL Assay diluent A (AdA) from the BDNF ELISA kit (Mature BDNF rapid^™^, Biosensis, Australia) to achieve a 1:5 dilution. This resulting solution (325 μL) was used for BDNF ELISA according to the kit protocol. With this volume, triplicate measurements were possible (100 μL per well).

For GDNF, only 10 μL of clear supernatant were mixed with additional 10 μL of RIPA buffer in a separate tube to be used for BCA (20 μL, dilution 1:2), since the procedure allows for using only 10 μL per well when necessary (see the BCA protocol below). The reason for this is that GDNF content in the brain is much smaller than that of BDNF and thus it is advised to use as much of undiluted sample as possible. Hence, the remaining 80 μL of clear supernatant were mixed with 120 μL of AdA (GDNF rapid^™^: rat, Biosensis, Australia) to achieve a 1:2.5 dilution, which was shown to yield the most robust results for GDNF. However, only duplicate measurement was possible due to the limitations of resulting volume (200 μL).

All further steps were performed according to manufacturer’s instructions from the respective ELISA kits. 100 μL of diluted standards, QC samples, experimental samples and blanks were added to pre-coated microplate wells in triplicate (BDNF) or duplicate (GDNF), sealed and incubated on a plate shaker (140 rpm) for 45 min (BDNF) or 90 min (GDNF). After washing the plate 5 times with the wash buffer, 100 μL of detection antibody was added to the wells and the sealed plate was again incubated on a shaker for 30 min (BDNF) or 60 min (GDNF). Following the next wash, 100 μL of streptavidin-HRP conjugate were added to the wells and incubated for 30 minutes. After the third wash, 100 μL of TMB was added to the wells and incubated for 8 min (BDNF) or 30 min (GDNF) without shaking in the dark. Before the final step, absorbance at 650 nm was read on a microplate reader (BioTek Epoch2) to determine if the optical density (OD) has reached the optimum for stopping the reaction (~ 1.2–1.3 for BDNF and ~ 0.9–1.1 for GDNF). Finally, to stop the reaction, 100 μL of stop solution was added to the wells and OD was measured at 450 nm.

BCA assay was performed according to manufacturer’s instructions using Pierce^™^ BCA Protein Assay Kit (ThermoScientific, Germany). For BDNF samples, 25 μL of 1:2 RIPA buffer dilution were pipetted into the microplate well (duplicates) and mixed with 200 μL of the working reagent (50:1, Reagent A:B), bringing the sample to working reagent ratio to 1:8. For GDNF samples, we mixed 10 μL (duplicates) of 1:2 RIPA buffer dilution with the same 200 μL of working reagent (WR), resulting in 1:20 sample:WR ratio. After the addition of the working reagent, the plate was sealed and kept on a plate shaker for 30 seconds, before being transferred into BioTek Epoch2 plate reader and incubated at 37 °C for 30 min. Absorbance was measured after cooling the plate to room temperature at 562 nm. For analysis, average absorbance of blank replicates was subtracted from that of all individual standard and sample replicates and used to prepare the standard curve and determine the protein concentration using sigmoidal 4-parameter logistic curve-fitting algorithm (GraphPad Prism). Results were multiplied by dilution factors and normalized to total protein content in each sample measured in BCA assay.

### Voluntary choice home cage drinking.

Experimental design from operant self-administration was adjusted to the conditions of home cage drinking. Male (n = 23) and female (n = 24) ZI Wistar rats were given continuous access to 10% alcohol bottles in their home cage, thus consuming alcohol under a two-bottle free-choice paradigm for 4 weeks. Both bottles were equipped with special bottle caps that minimized spillage and evaporation. The positions of water and alcohol bottles were changed weekly to minimize position preference. On the week of aversion resistance test, 0.025 g/L quinine HCL was added to the 10% alcohol bottles and was presented to the animals at the start of the active (dark) phase of circadian cycle for two consecutive days, while quinine-free alcohol was given at the end during inactive (light) phase and remained available for two full days after the test. Oxa-noribogaine (30 mg/kg) or vehicle were administered intraperitoneally 2 hours after the end of the first quinine-adulterated phase. Consumption of each type of alcohol was measured by weighing the bottles before and after each phase and converted into g/kg/day intake according to the following formula:

$$\frac{\left(\frac{{\Delta m}_{b(g)}}{\rho_{10\%}}\times 0.1\times \rho_{100\%}\right)}{m_{a(kg)}}$$

Where Δm_b(g)_ – 10% EtOH bottle weight difference between t0 and t1 in grams, _10%_ – density of 10% ethanol solution (v/v) (0.98476 g/mL), _100%_ – density of 100% ethanol (0.7893 g/mL), m_a(kg)_ – body mass of the animal in kg.

To assess the effects of oxa-noribogaine on relapse-like drinking, ZI Wistar rats were tested in alcohol deprivation effect (ADE) model. After the initial period of 8 weeks of continuous access to alcohol-containing solution, these bottles were removed and the rats underwent a first period of deprivation that lasted for one week. 10% alcohol solution-containing bottles were reintroduced at the end of deprivation week and rats continued drinking for another 4 weeks before the next cycle of deprivation began. Each week, fresh bottles of water and 10% EtOH were prepared and total liquid consumption was measured by weighing each bottle on the scales before being presented to the rats and after 7 days of continuous consumption. During the weeks before and after the deprivation, bottles were weighed every day and thus daily consumption was measured. Two cycles of drinking and deprivation were introduced before the drug testing; rats were randomly assigned to control or treatment groups based on the alcohol intake values (g/kg/day) during the baseline (= preceding deprivation) week so that the means between groups did not differ from each other for more than 0.3 g/kg/day. Oxa-noribogaine (30 mg/kg) was applied intraperitoneally with the total volume of injection kept at 2 mL/kg body weight. The application was done on the last day of the second deprivation phase 24h before the reintroduction of alcohol-containing bottles, and daily consumption of water and alcohol was measured for the following 5 days. Alcohol deprivation effect was defined as relapse size, calculated as the difference in total alcohol intake (delta) in g/kg/day between the first day of deprivation and average value at baseline. Male (n = 40) and female (n = 40) rats of heterogeneous background (NIH) underwent identical experimental designs as outlined above for ZI Wistar rats in a multi-site experiment involving research sites in Berlin, Erlangen and Mannheim using the preRCT design for rigorous research practice as previously published^59^.

### Statistics.

Graphs were created and statistical tests were applied using GraphPad Prism, Version 10. For comparisons among three or more groups, one-way analysis of variance (ANOVA), two-way ANOVA, or two-way repeated measures ANOVA was used, where applicable. When a significant main effect was detected, a post hoc analysis with Tukey’s multiple comparisons test, Holm-Sidak multiple comparisons test, or Dunnett’s multiple comparisons test w. For comparisons between two groups, the parametric two- tailed unpaired or paired t-test was used, where applicable. Detailed statistical analyses including the sample size are indicated in the figure legends and methods subsection for each experiment. Statistical significance was set at p < 0.05. Statistical analyses were performed using GraphPad Prism 7.04 (GraphPad Software, San Diego, CA).

## Supplementary Material

Supplementary Files

This is a list of supplementary files associated with this preprint. Click to download.

- SupplementaryInformationSynthesisMALDI.pdf

## Data Availability

All data and materials used in the analysis are be available upon reasonable request.
